# Tert-butyldimethylsilyl chitosan synthesis and characterization by analytical ultracentrifugation, for archaeological wood conservation

**DOI:** 10.1007/s00249-020-01450-z

**Published:** 2020-08-26

**Authors:** Jennifer M. K. Wakefield, Susan Braovac, Hartmut Kutzke, Robert A. Stockman, Stephen E. Harding

**Affiliations:** 1 National Centre for Macromolecular Hydrodynamics (NCMH), School of Biosciences, University of Nottingham, Sutton Bonington, Nottingham, LE12 5RD UK; 2 School of Chemistry, University of Nottingham, University Park Nottingham, Nottingham, NG7 2RD UK; 3 Museum of Cultural History, University of Oslo, St. Olavs plass, Postboks 6762, 0130 Oslo, Norway

**Keywords:** Analytical ultracentrifuge, NMR, Combined molar mass method

## Abstract

The Oseberg ship is one of the most important archaeological testimonies of the Vikings. After excavation in 1904, the wooden gravegoods were conserved using alum salts. This resulted in extreme degradation of a number of the objects a hundred years later through acid depolymerisation of cellulose and lignin. The fragile condition of the artefacts requires a reconsolidation which has to be done avoiding water as solvent. We synthesized *tert-*butyldimethylsilyl (TBDMS) chitosan which is soluble in a 50:50 solution of ethyl acetate and toluene. Measurement of its molecular weight, to anticipate its penetration, provided a challenge as the density difference of the polymer and solvent was too small to provide adequate solute redistribution under a centrifugal field, so a two-stage process was implemented (i) determination of the weight-average molar mass of the aqueous soluble activated precursor, chitosan mesylate, *M*_w,mc_ using sedimentation equilibrium with the SEDFIT-MSTAR algorithm, and determination of the degree of polymerisation *DP*; (ii) measurement of the average degree of substitution *DS*_TBDMS_ of the TBDMS group on each chitosan monosaccharide monomer unit using NMR, to augment the *M*_w,mc_ value to give the molar mass of the TBDMS-chitosan. For the preparation, we find *M*_w_ = 9.8 kg·mol^−1^, which is within the acceptable limit for penetration and consolidation of degraded wood. Future work will test this on archaeological wood from different sources.

## Introduction

The Oseberg collection is in dire need of re-conservation. The Oseberg Viking ship burial was discovered in 1903 and excavated in 1904 (see for example Braovac et al. 2018). The grave mound contained a rich collection of different artefacts; amongst them, many wooden objects which are today displayed at the Viking Ship Museum in Oslo, Norway (www.khm.no). The most degraded wooden artefacts were treated in 1905 with concentrated solutions of alum salts [KAl(SO_4_)_2_.12H_2_O and NH_4_Al(SO_4_)_2_.12.H_2_O] heated to 90 ℃. Although this treatment allowed the objects to retain their shape upon drying, it has caused degradation in the long term, as the hot alum treatment produced sulfuric acid which was absorbed by the wood, and which exhibits today an average pH of about 2–2.5 (Braovac and Kutzke 2012). This sulfuric acid causes hydrolytic cleavage of the *β* (1 → 4) glycosidic bond in cellulose and breaks down the cellulose in the wood. There is now very little cellulose left and the lignin in the wood is also heavily oxidised (Braovac and Kutzke 2012; Fors and Sandström 2006; McQueen et al. 2017; Smidsrød et al. 1966). The wood is now very fragile and treatment is required to preserve the artefacts for future generations.

The presence of acid in the wood is of great concern. Since the water-soluble alum supports the remaining wood structure, de-acidification by immersion in water could cause the complete collapse of the most degraded artefacts. Therefore, a non-aqueous method is required for the re-treatment of these objects. There are a few non-aqueous treatment methods for wood and they are all derived from fossil fuels (Kučerová 2012). There has, in addition, been a drive in recent years to try to use more natural-based polymers (Cipriani et al. 2010; McHale et al. 2017) rather than petrochemical-based synthetic materials.

Chitosan, a *β* (1 → 4) glucan derived from the shells of crabs, lobsters, and other crustaceans, has been selected for its molecular similarity to cellulose in the wood and, as it is a waste product of the canning industry, it is cheap to obtain and sustainable (Ravi Kumar 2000; Younes and Rinaudo 2015). Chitosan has previously been investigated as an aqueous treatment for conservation (Christensen et al. 2015; Walsh et al. 2017). Chitosan in itself is not soluble in organic solvents; it is only soluble in acidic aqueous solutions. Therefore, the polymer requires modification to make it soluble in a suitable organic solvent. These modifications should avoid the use of ester bonds which would be prone to break down particularly in an acidic environment, such as alum-treated wood (Mourya and Inamdar 2008).

**Fig. 1.**
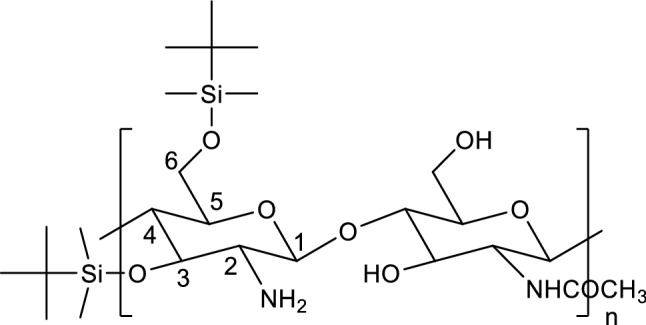
3,6-Silylated chitosan

We decided to take advantage of a procedure involving mesylation developed some years ago by Rúnarsson et al. (2008) who have made an organic solvent-soluble chitosan derivative, *tert-*butyldimethylsilyl (TBDMS) chitosan (Rúnarsson et al. 2008) with the TBDMS group being added at both carbon atoms 3 and 6 on the pyranose chains (Fig. 1).

Good solubility [i.e., completely soluble at 10% (w/v)] was found by Rúnarsson et al. (2008) for chitosan-TBDMS oligomers with a degree of substitution (DS) of 2.2 in a range of different organic solvents such as N-methyl-2-pyrrolidone (NMP), dimethylformamide (DMF), dimethylsulfoxide (DMSO) diethyl ether, triethylamine, pyridine, tetrahydrofuran (THF), acetone, 1-butanol, 2-propanol, ethyl acetate, ethanol, dichloromethane (DCM), and chloroform (Rúnarsson et al. 2008). A DS of 1.29 showed reduced solubility in diethyl ether, acetone, and ethanol. The chitosan-TBDMS polymer with a DS of 1.94 had reduced solubility in all solvents, but was still completely soluble at 2.5% in pyridine, 1-butanol, 2-propanol, and ethyl acetate. Further optimisation by the Runarson et al. team and Song et al. (2010) resulted in a 90% yield for the mesylate and 96% yield for the 3,6-di-O-TBDMS-chitosan polymer. The polymer becomes ‘superhydrophobic’ (Wang and Jiang 2007) and this ‘superhydrophobicity’ may help to prevent swelling and subsequent shrinkage of the wood.

In this study we therefore, use the Rúnarsson et al. (2008)/Song et al. (2010) method to produce TBDMS-substituted polymeric chitosan as an organic soluble-modified chitosan for potential consolidant use. For our study, toluene proved a useful solvent for this material which permitted full solubility for the TBDMS chitosan at the sort of scale-up concentrations and volumes which would be useful for potential archaeological wood conservation. For linear PEG polymers used in conservation treatments where aqueous formulations are allowed (e.g., treatment of wood from the Vasa and Mary Rose), it was found that a molar mass of ~ 0.2–4.0 kg·mol^−1^ was useful, and we have recently found that aqueous soluble chitosan of a similar molar mass (~ 5.0 kg·mol^−1^) gives penetration (Wakefield et al. 2018). The non-aqueous analogue, TBDMS chitosan, could be bulkier but shorter which may allow the molar mass to be higher than that of PEG. A backbone of 5.0 kg·mol^−1^ seemed reasonable to ensure that it penetrates into the wood. If the polymer is too small, it will not have a sufficient consolidation effect.

As has been discussed before (Wakefield et al. 2018; Harding 2018) for characterizing molar masses of consolidants, the matrix-free and absolute method of sedimentation equilibrium in the analytical ultracentrifuge is particularly attractive. Measurement of the molar mass of a potential consolidant substance with sedimentation equilibrium, however, provides a challenge as the density difference of the TBDMS chitosan and toluene was too small to provide adequate solute redistribution under a centrifugal field: so a two-stage process was implemented.

First, we determine the weight-average molar mass *M*_w_ of the aqueous soluble precursor, chitosan mesylate (Mes-CS), *M*_w,mc_, and hence the degree of polymerisation (DP) using sedimentation equilibrium and the SEDFIT-MSTAR algorithm of Schuck, Harding, and coworkers (Schuck et al. 2014). Second, we take the value for the average degree of substitution *DS*_TBDMS_ of the TBDMS group onto the chitosan monomer estimated using nuclear magnetic resonance spectroscopy (in the ethyl acetate/toluene solvent) and multiply this by the degree of polymerisation of Mes-CS, finally correcting for the removal of the mesylate.

## Materials and methods

### *Generation of a low molar mass (*~ *5 kg·mol*^*−1*^*) chitosan template*

We follow the oxidative degradative procedure in the presence of ultra-violet radiation of Wakefield et al. (2018) based on an earlier procedure of Wang et al. (2005). Using chitosan (22.0 g) from Norwegian Chitosan Ltd (Gardemoen, Norway) with a degree of acetylation *DA* = 0.1, a 4% (40 g/l) solution was prepared by dissolving (with stirring) chitosan in 0.2% acetic acid for 1 h yielding a clear yellow solution. Hydrogen peroxide was then added (0.2% after dilution) and the solution was exposed to UV light for 1 h at 20.0 °C followed by neutralization with 1 M sodium hydroxide: this causes the chitosan to precipitate out of solution. This solution was then centrifuged and the solid product was washed with deionised water (3 × 50 mL) and centrifuged each time (10,000 rpm for 5 min). The solid product was frozen in an − 80.0 °C freezer overnight and freeze dried. The five batches yielded a total recovery of (73.3 ± 4.0)%.

### Generation of chitosan mesylate (Mes-CS)

Prior to the reaction with tert-butyl dimethyl silyl chloride (TBDMSCl), ‘intermediate’ form of chitosan (chitosan mesylate) was produced facilitating dissolution in dimethyl sulfoxide (DMSO) without compromising the amino group. We use a procedure given by Song et al. (2010) adapted to minimize chain depolymerisation through acid exposure, and use isopropanol rather than ethanol followed by acetone for precipitation. The chitosan (20.0 g, F.W. 165.36, 121 mmol) was suspended in water (130 mL) and placed in a 10 °C ice bath. 15 mL of methanesulfonic acid (CAS 75–75-2, Alfa Aesar, A13565 lot 10,198,453) was then added slowly, leaving a clear solution. After leaving for 1 h, the product was precipitated after the addition of isopropanol: then filtered and washed twice with isopropanol and then washed with acetone. The product was then filtered once again and left to air dry for 1 h after which the product was re-dissolved in deionised H_2_O (15 mL). It was then re-precipitated in acetone (450 mL) and washed with acetone (3 × 125 mL). The resulting white powder was then air dried/vacuum dried (27.83–28.23 g, yield 91.2–92.7%, 111–112 mmol). Its purity was established by solid-state ATR-FTIR and ^1^H NMR at 400 MHz—which established a degree of substitution *DS*_mesylate_ ~ 1 of mesylate. The details of the FTIR and ^1^H NMR are as follows, where the notations have their usual meaning (see, for example, Rúnarsson et al. 2008; Song et al. 2010): FTIR: v 3359 (B), 2936 (m), 1635 (vs), 1526 (vs), 777 cm^1^. ^1^H NMR (400 MHz, D2O) δ 2.8 (s), 3.2 (m), 3.8–3.9 (m), 4.8 (s).

### Generation of tert-butyl dimethyl silyl (TBDMS) chitosan

We follow essentially the procedure of Song et al. (2010) and Rúnarsson et al. (2008). Chitosan mesylate, Mes-CS (12 g, 48 mmol), was re-dissolved in dry DMSO (120 mL), stirring under nitrogen. Imidazole (32.47 g, 477 mmol 5 × excess) and tert-butyl dimethyl silyl chloride (TBDMSCl) (35.93 g, 238 mmol, 2.5 × excess), were dissolved in dry DMSO (140 mL) under nitrogen. This was then added dropwise to the chitosan mesylate at room temperature still under nitrogen. After 2 h, dry toluene (140 mL) was added and left stirring under nitrogen for another 22 h. Deionised H_2_O (100 mL) was then added and an emulsion formed, which was extracted with a solution of ethyl acetate (100 mL) and toluene (50 mL), and then a further ethyl acetate application (3 × 75 mL). Ethyl acetate rather than hexane (Rúnarsson et al. 2008) as it facilitated better dissolution. The ethyl acetate fraction was then washed with saturated NaCl (3 × 50 mL). The ethyl acetate was dried over sodium sulfate and the latter was filtered off and the ethyl acetate concentrated on the rotary evaporator until ~ 30 mL was left. The product then precipitated in acetonitrile (300 mL) and washed with acetonitrile (4 × 75 mL). This was dried in the fume hood overnight and then dried in a 40.0 ℃ vacuum oven to give a white powder (13.27–16.02 g, yield 65.0–78.5%). The high purity of the product was verified by ATR-FTIR, and the degree of substitution DS evaluated by ^1^H NMR spectroscopy at 400 MHz. The details of the FTIR and 1H NMR are as follows (Rúnarsson et al. 2008; Song et al. 2010): v, 2953–2856 (s), 1689 (vs), 1251, 833, 775 (vs) cm^−1^. ^1^H NMR (400 MHz, CDCl_3_) d 0.08 (br s), 0.89 (br s), 1.98 (br s), 2.72 (br s), 3.3–3.89 (m), 4.29–4.40 (m), 8.11 (br s).

### Sedimentation equilibrium in the analytical ultracentrifuge

A Beckman XL-I analytical ultracentrifuge (AUC) equipped with Rayleigh interference optics was used. 20 mm optical path length cells with volumes of 145 μL were used for the analysis of the depolymerised chitosan and 12 mm optical path double sector cells were employed for the chitosan mesylate analysis: solution and solvent (buffer) reference channels were filled to 100 μL which gave short solution columns, facilitating equilibrium in ~ 24 h. A high rotor speed of 40,000 rpm (appropriate for material of the expected size range) at a temperature of 20.0 °C. Scans with Rayleigh interference optics were taken every hour until equilibrium was reached: this was assessed using the SEDFIT-Tools-Test approach to equilibrium (courtesy of P. Schuck). Analysis was carried out using SEDFIT-MSTAR (Schuck et al. 2014) which provides the (apparent) weight-average molar mass *M*_w,app_ [obtained using the hinge point method—see (Schuck et al. 2014)]. The hinge point proved more appropriate for these samples than the M* method of Creeth and Harding (1982) because of their high polydispersity. Loading concentrations from 0.30 to 1.0 mg/mL for the chitosan with long path length cells and 0.4–1.0 mg/mL for short path length cells were employed to monitor for any associative or non-ideal effects, which were negligible. Chitosan which had been depolymerised as described before (Wakefield et al. 2018) was dissolved in 0.2 M acetate buffer and the chitosan mesylate in a 0.10 M phosphate-buffered solution supplemented with NaCl to an ionic strength of 0.10 (Green 1933). A value for the partial specific volume (ῡ) of 0.57 mL/g was used (Morris et al. 2009).

### Treatment of artificially degraded wood

Birch wood staves were obtained from Norwegian food stores. They were of standard size and shape, ~ 18 cm long and 1 × 1 cm in cross section with masses from 9 to 11 g. A representative number of birch staves were identified in the light microscope to confirm wood genus (birch, Betula, spp.). The wood was initially waterlogged then immersed in treatment solutions. The birch was then degraded using two solutions a 5% sulfuric acid solution (vol/vol) to degrade the holocellulose fraction and a 5% sodium hydroxide solution (w/vol) which would cause lignin and hemicellulose degradation. The combined degradation caused by these solutions would also increase porosity in the wood and thus increase degradation rate. The approximate liquid (L, in litres)-to-solid (S, in kilograms) ratio used for experiments was calculated based on the starting volumes of 1.8 L. The 20 staves gave a total of 176 g and an L/S ratio of 10.2. The acid solution was heated to 90.0 °C to increase degradation rate (the approximate temperature used during the original alum treatment of Oseberg wood after excavation in 1904) using a water bath. The sodium hydroxide solution was kept at room temperature. Soaking times in each solution lasted for approximately 6 days. Before transferring from one solution to the other, the samples were rinsed in several changes of tap water. After removal from the final degradation bath, a longer period of rinsing was undertaken. The final rinse water pH was ~ 7.0. The wood had a total time of 4784 h in acid, where 295 h were at 90 °C, and 1006 h in base. The wood was then freeze dried for 7 days. The wood density dropped by (55.4 ± 1.2)% on average to (0.382 ± 0.040) g/mL. Samples were then cut into 1 cm cubes (3 for each treatment and 2 for each control) and then soaked overnight in 50:50 ethyl acetate and toluene followed by transfer to a 10% TBDMS chitosan in 50:50 ethyl acetate and toluene solution. Controls were left in the solvent. After 2 weeks the cubes were taken out, the surfaces cleaned with a cotton bud loaded with the solvent. The wood pieces were air dried. The photographs, weight, and dimensions were taken before and after this process.

To determine if the polymers penetrated into the wood, scanning electron microscopy coupled with energy-dispersive X-ray spectroscopy (SEM–EDS) was used to map the silicon element present in the polymer. The instrument was an FEI Quanta 450 Scanning Electron Microscope coupled with an Oxford X-Max^N^ 50 mm^2^ detector, using low vacuum mode at (70pa). Spot size 6, HV 15.00 kV, working distance 10.4 mm.

## Results

### Purity and degree of substitution (DS)

**Fig. 2:**
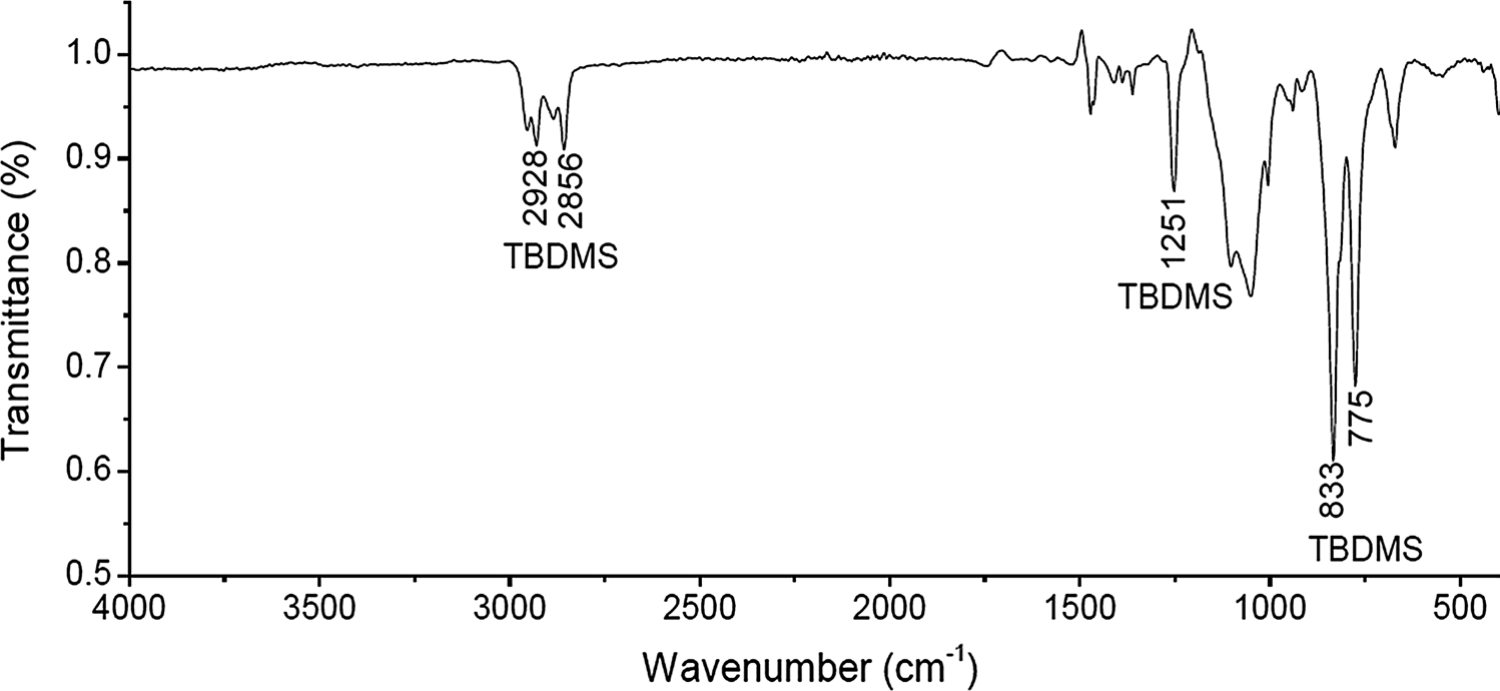
Solid-state attenuated total reflection Fourier transform infra-red (ATR-FTIR) spectrum of TBDMS chitosan

The purity/chemical integrity of the substituted material, i.e., the inclusion of the TBDMS groups on the chitosan backbone was confirmed by Attenuated Total Reflection Fourier Transform Infra-Red Spectroscopy (ATR-FTIR) wherein diagnostic signals for a silyl group were noted (Fig. 2).

**Fig. 3.**
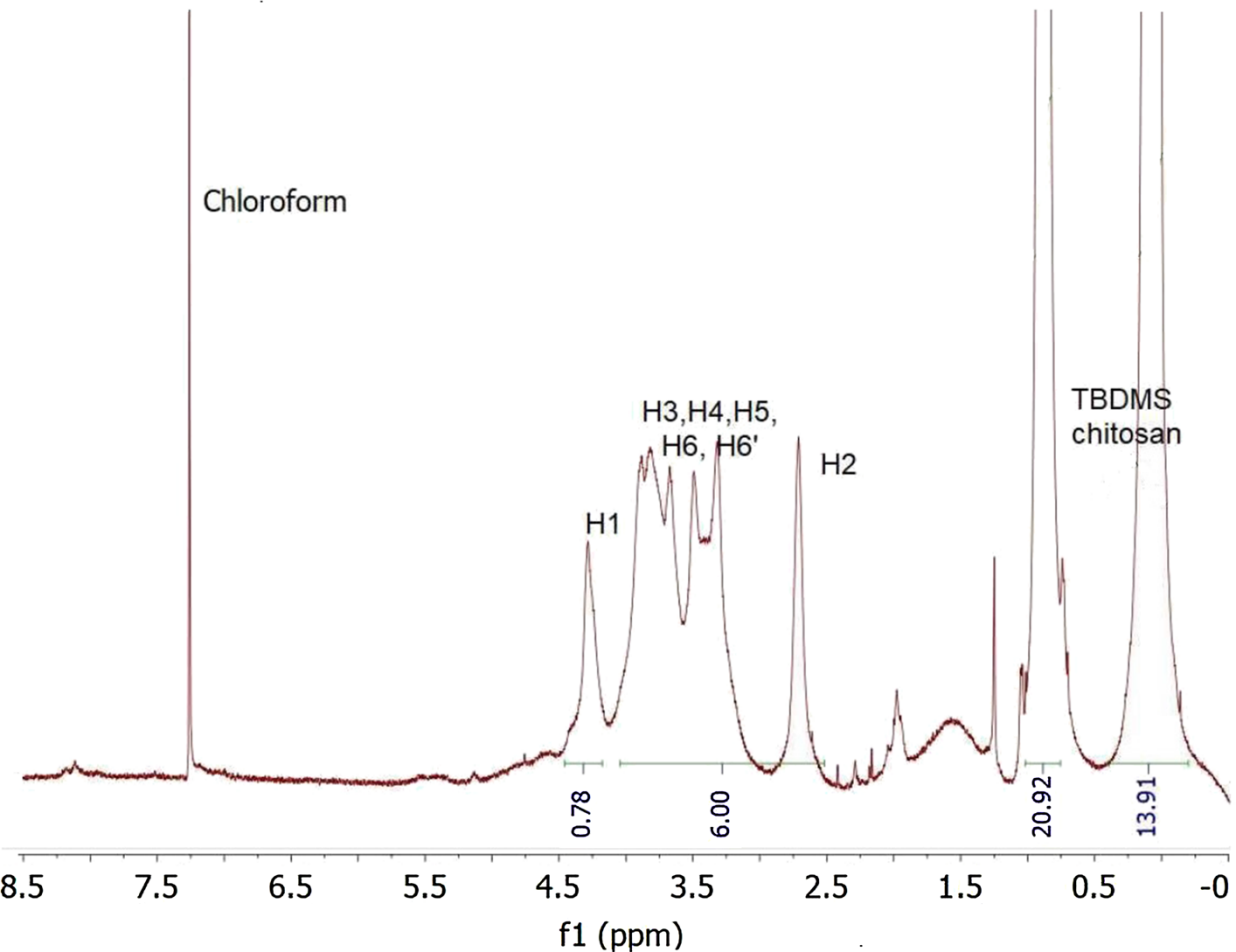
^1^H NMR analysis—intensity versus frequency (concentration) f1 in parts per million ppm—of TBDMS chitosan in deuterated chloroform. Integration of the appropriate peaks reveals a degree of substitution *DS*_TBDMS_ = 2.3

Then, using ^1^H NMR conducted in deuterated chloroform, we determined the degree of substitution, *DS*_TBDMS_ for TBDMS—see Fig. 3. This is found from the ratio of the NMR integral of either set of the hydrogens from the butyl groups of TBDMS chitosan divided by integrals of H2, H3, H4, H5, H6, and H6’ from the chitosan backbone taking into account the number of protons using the formula given by Rúnarsson and colleagues: DS=([(CH)_3_]/[H-2–H-6’] × (6/9)) (Rúnarsson et al. 2008). This gave a *DS*_TBDMS_ of ~ 2.3.

### Molar mass of chitosan and the chitosan mesylate intermediate

**Fig. 4:**
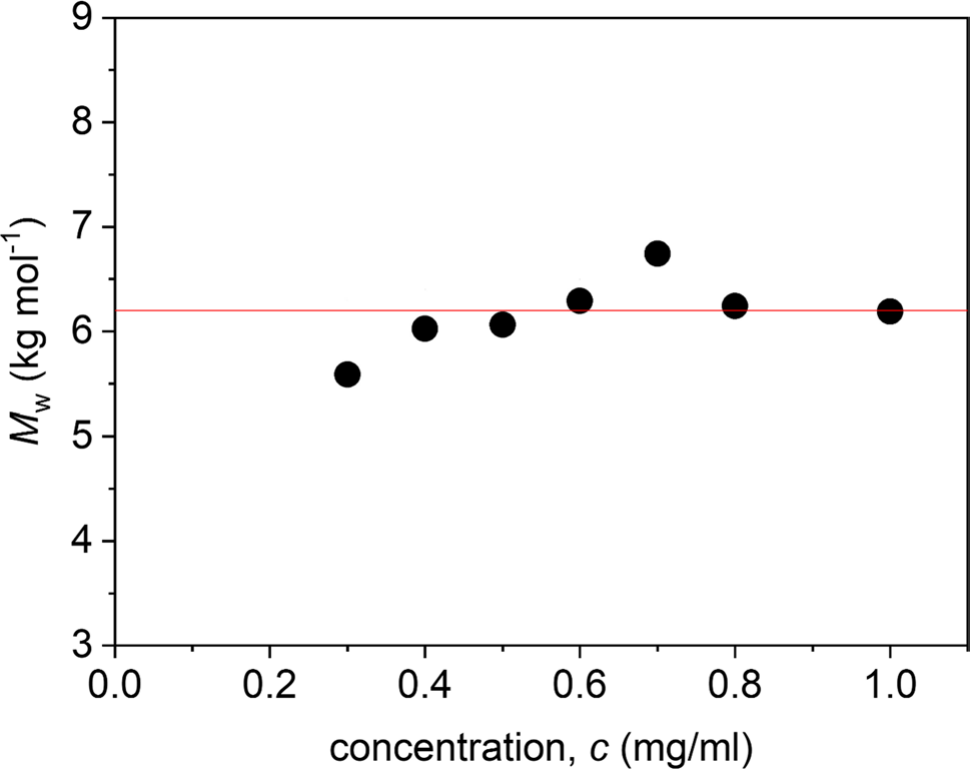
Plot of *M*_w,app_ from SEDFIT-MSTAR analysis (using the hinge point method) vs loading concentration, *c* for the scaled-up preparation of depolymerised chitosan run at 40,000 rpm in *I* = 0.10 phosphate-chloride buffer. Non-ideality is negligible over the concentration range studied with *M*_w_ ~ *M*_w,app_ = (6.2 ± 0.3) kg·mol^−1^

Depolymerisation was previously shown to reduce the (weight average) molar mass of an earlier preparation of kitnor chitosan from a weight average molar mass *M*_w_ of (14.2 ± 1.2) kg·mol^−1^ to (4.9 ± 0.7) kg·mol^−1^ (Wakefield et al. 2018). We followed this work by scaling up the production in five batches of 22 g. These five batches were combined before chemically modifying the polymer. From Fig. 4, it can be seen that the molar mass of the combined batch is *M*_w_ = (6.2 ± 0.3) kg·mol^−1^. This is an acceptable molar mass as a starting point to develop a conservation material (Wakefield et al. 2018).

**Fig. 5:**
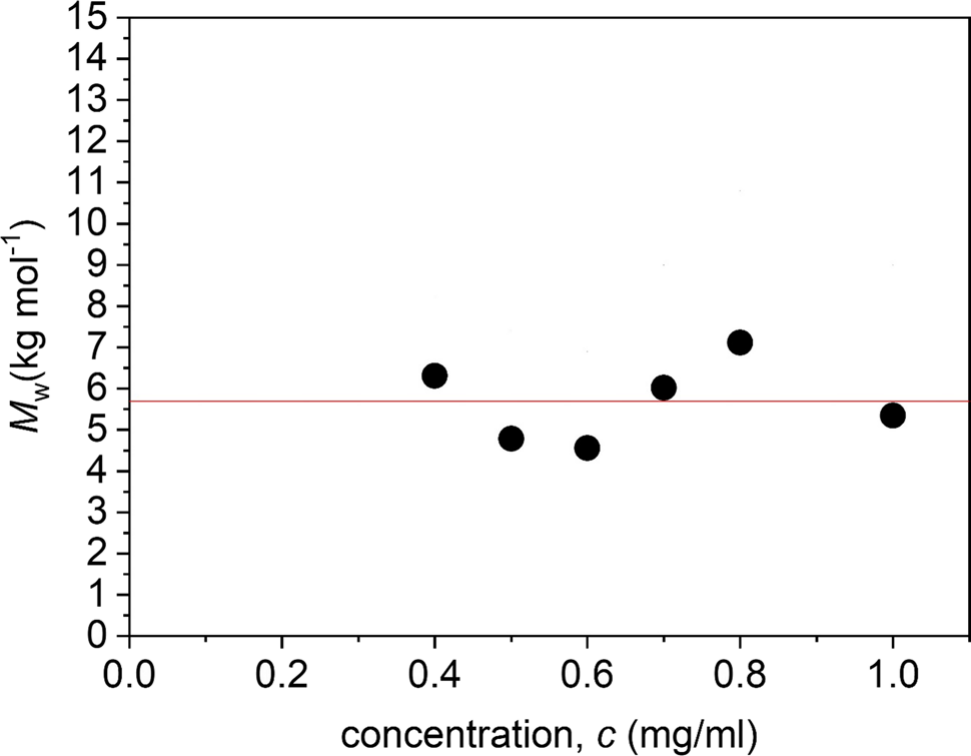
Plot *M*_w,app_ from SEDFIT-MSTAR (estimated from the hinge point) vs loading concentration, *c* for chitosan mesylate run at 40,000 rpm. Non-ideality is negligible over the concentration range studied with *M*_w_ ~ *M*_w,app_ = (5.7 ± 1.0) kg·mol^−1^. All samples were run at an equilibrium rotor speed of 40,000 rpm. *I* = 0.10 M phosphate chloride at 20.0 ℃

**Fig. 6:**
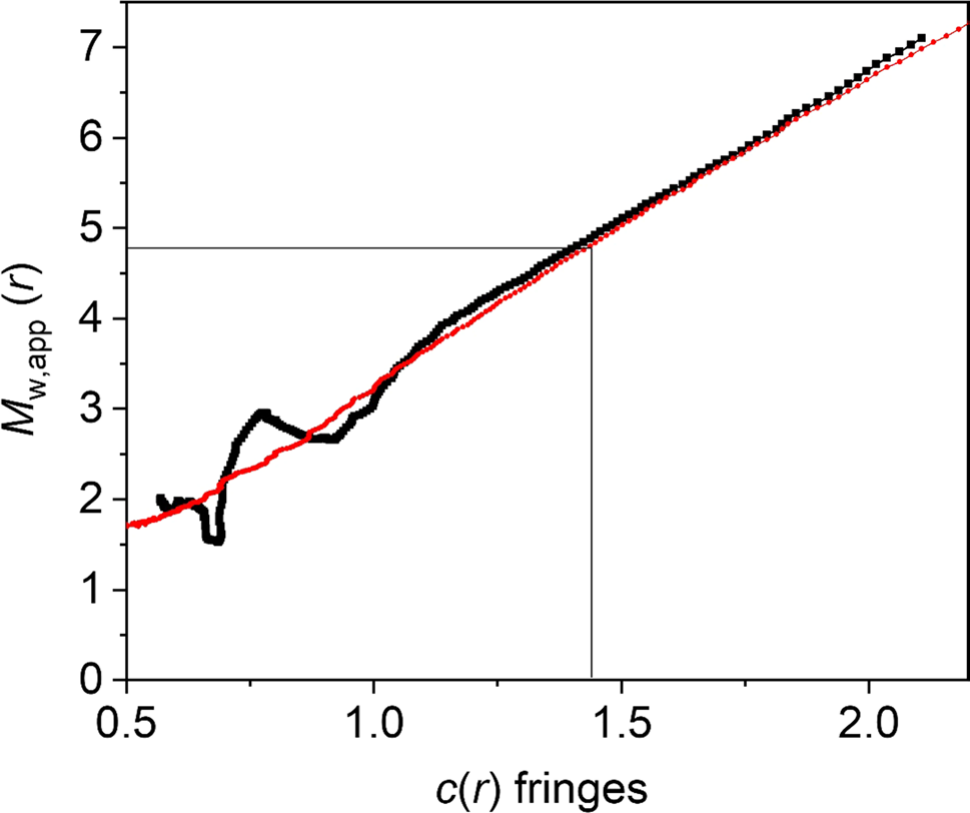
Point average molecular weights *M*_w_(*r*) (in kg/mol) as a function of local concentration *c*(*r*) in the centrifuge cell (in fringe numbers) at different radial positions *r* show considerable polydispersity. SEDFIT-MSTAR analysis of chitosan mesylate at *c* = 0.5 mg/mL. Other information as Fig. 5. The plot also shows the hinge point where the local *c*(*r*) = the initial loading concentration *c*. *I* = 0.10 M phosphate chloride at 20.0 ℃

Following modification to chitosan mesylate (Mes-CS)—which helps control the later addition of TBDMS—further sedimentation equilibrium measurements showed that the (weight average) molar mass *M*_w_ was (5.7 ± 1.0) kg·mol^−1^ (Fig. 5)—corresponding to a degree of polymerisation *DP* ~ 22.6—and showing considerable polydispersity (Fig. 6). SEDFIT-MSTAR analysis (Schuck et al. 2014) offers also an estimate for the z-average molar mass *M*_z_ as well as *M*_w_, and this leads to an estimate for the polydispersity (in terms of *M*_z_/*M*_w_) of ~ 1.9 for chitosan mesylate compared with ~ 1.2 for the untreated chitosan.

### Evaluation of the molar mass of TBDMS chitosan

The final step in the molar mass evaluation is to take the value for the degree of substitution of the hydroxyls in a chitosan monosaccharide monomer unit with a TBDMS group determined using ^1^H nuclear magnetic resonance to calculate the weight-average molar mass of TBDMS chitosan using:

1 $$M_{{\text{w}}} = M_{{\text{w,mc}}} + \left( {DP \times DS_{{\text{TBDMS}}} \times M_{{{\text{TBDMS}}}} } \right) - \left( {DP \times DS_{{\text{mesylate}}} \times M_{{{\text{mesylate}}}} } \right)$$

From the degree of substitution of TBDMS *DS*_TBDMS_ of 2.3 (Fig. 3), an average degree of polymerisation *DP* ~ 22.6,  molar mass of chitosan mesylate of 5.7 kg·mol^−1^, and correcting for the removal of the mesylate which had a degree of substitution *DS*_mesylate_ of ~ 1.0, we obtain a value of *M*_w_ = 9.8 kg·mol^−1^ for TBDMS chitosan.

### Accuracy of the DS determination by ^1^HMR spectroscopy

We have followed the Rúnarsson et al. (2008) method for estimating the average DS of TBDMS residues onto the chitosan molecule. Rúnarsson et al. (2008) do not give the errors/accuracy of this method. However, a very detailed comparison has been given by Hadi et al. (2020) of ^1^H NMR and stoichiometric methods for DS determination of two other polysaccharides—rice and quinola starches, with different degrees of aceylatation, propionylation, and butyrylation. 24 samples were analyzed in total, and agreement to within (9.4 ± 1.4)% was obtained. The additional molar mass due to the TBDMS substitution is, therefore, estimated to be (3. 8 ±  0.3) kg/mol leading to an estimate for the molar mass for the intact TBDMS chitosan by this “hybrid” sedimentation equilibrium–^1^H NMR method of (9.8  ±  1.5) kg/mol.

### Potential of Di-TBDMS chitosan for wood conservation

**Fig. 7:**
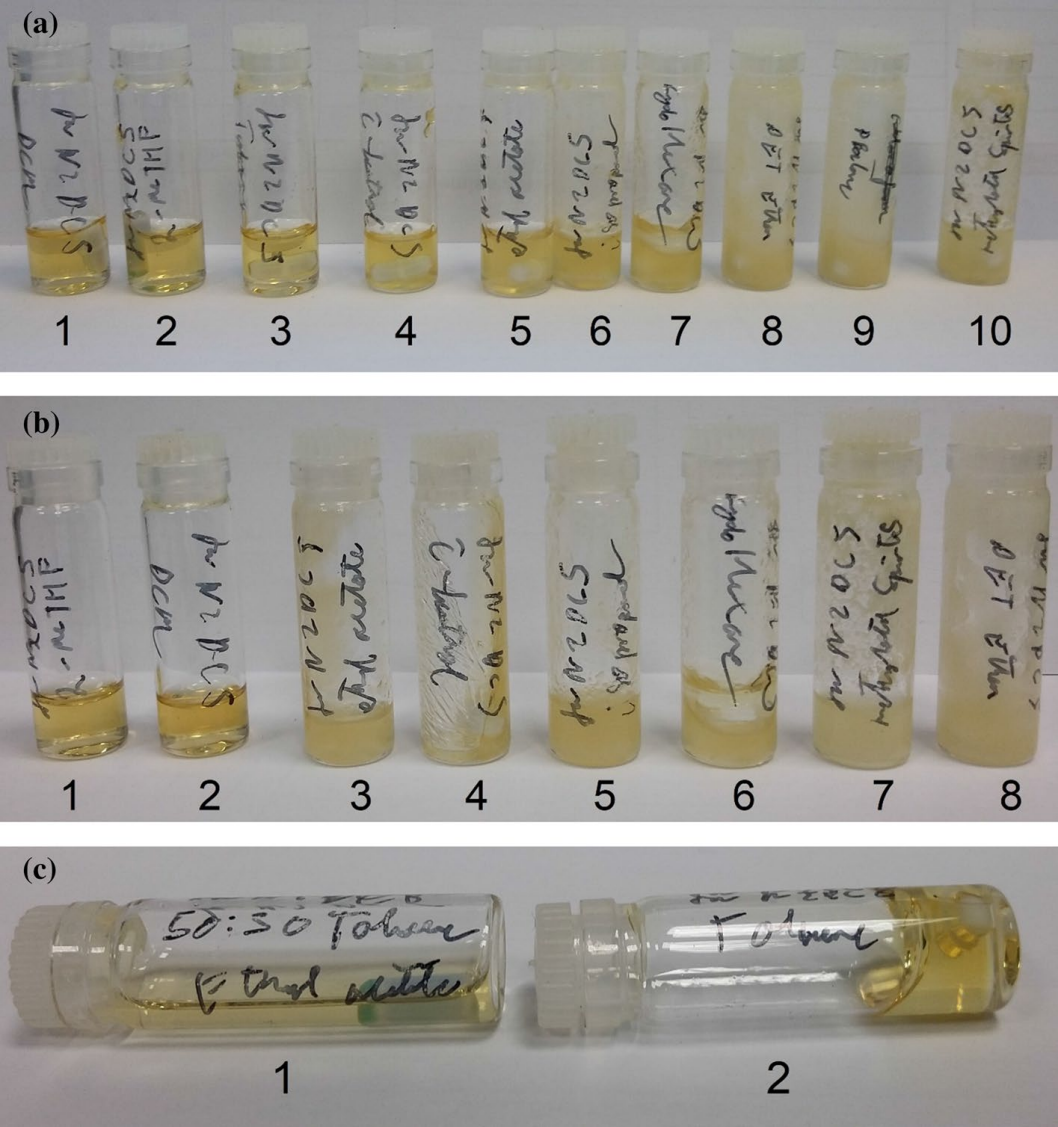
Comparison of solubilities of 10% TBDMS chitosan (w/v) in (**a**) 1 DCM, 2 2-meTHF, 3 toluene, 4 t-butanol, 5 ethyl acetate, 6 isopropanol, 7 cyclohexane, 8 PET ether, 9 pentane, and 10 methylated spirits. **b** 6 days later in 1 2-meTHF, 2 DCM, 3 ethyl acetate, 4 t-butanol, 5 isopropanol, 6 cyclohexane, 7 methylated spirits, and 8 PET ether. There is reduced solubility after 6 days in ethyl acetate and isopropanol. **c** In toluene–ethyl acetate mixture (1) and toluene (2) with vessels tipped to show the lower viscosity of the toluene–ethyl acetate mixture. All measurements were taken at room temperature

Figure 7 compares the solubility of TBDMS chitosan in different solvents and shows how TBDMS chitosan starts to precipitate in some solvents over time. The low viscosity in a mixture of toluene and ethyl acetate would allow for archaeological wood application of TBDMS chitosan. Toluene has previously been used in wood conservation and has been found to cause less swelling in wood than polar solvents (Mantanis et al. 1994); however, the solution with TBDMS chitosan would be very viscous, preventing the material entering the cells. Ethyl acetate was previously investigated for wood conservation (McHale et al. 2016) in terms of sustainability and wood swelling and appears preferable to many other solvents. Adjusting the exact DS of TBDMS may allow ethyl acetate to be used alone.

### Treatment of artificially degraded wood

**Fig. 8:**
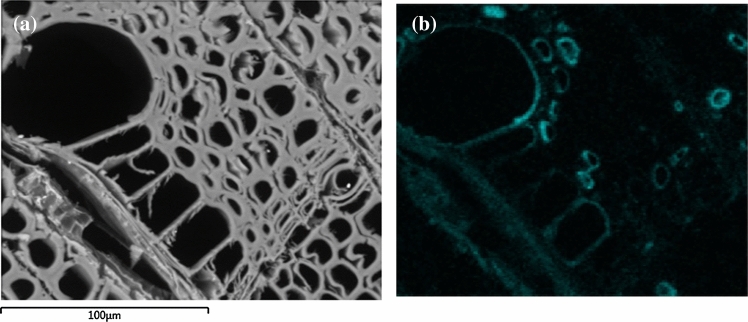
SEM–EDS images taken from the center of a 1 cm cube of artificially decayed birch treated with 10% TBDMS-chitosan in toluene/ethyl acetate. **a** Image showing the region used for elemental mapping. **b** EDS map of silicon

Treatment of artificially degraded wood with 10% TBDMS chitosan in toluene/ethyl acetate showed a weight gain of (19.6 ± 2.0)% after drying, while the solvent control increased by (8.0 ± 6.2)%. It is possible that not all the toluene evaporated at the time the samples were weighed. Volume increase of the treated wood by (3.8 ± 0.5)% was slightly greater than that for the controls, namely (2.1 ± 0.3)%, indicating that the treatment caused some swelling. SEM–EDS was carried out on the TBDMS chitosan-treated wood to determine its distribution by mapping for the silicon element in the polymer. Figure 8 shows that the TBDMS chitosan successfully penetrated the wood fully based on the presence of silicon in the core of the sample. SEM images also show that the polymer coats the cell walls rather than simply acting like a filler. This means that it would be easy to re-treat the sample without removing the TBDMS chitosan should it become necessary in the future.

## Conclusions

In this study, we have built on our previous study (Wakefield et al. 2018) successfully reducing the molar mass of chitosan and it making it soluble—to a high yield—in an organic solvent which is what we need for consolidating highly degraded alum-treated wood such as the now perilously fragile Oseberg artefacts from the Viking Ship Museum in Oslo. We have shown that using sedimentation equilibrium in the analytical ultracentrifuge combined with data from NMR, we can successfully avoid the problem of small density increments between polymer and solvent in determining the molecular weights of the promising TBDMS chitosan in a solvent of ethyl acetate and toluene. Our organic toluene/ethyl acetate soluble chitosan derivative was shown to be characterized by a (weight average) molar mass of ~  9.8 kg/mol, with a degree of polymerisation, *DP* of, 22.6 and degree of substitution by TBDMS residues of 2.3.

We then used this polymer to investigate its penetration into small degraded birch samples—which it successfully did, and it did not fill the cell lumens which allows a full re-treatment if necessary. The next step is to test the polymer’s effectiveness on archaeological wood.

For future consolidation, we are considering first deacidifying the wood with alkaline calcium hydroxide nanoparticles to raise the pH (Andriulo et al. 2018) and then adding the appropriate consolidant. Both steps of treatment will be conducted using an organic solvent, for the de-acidification as well for the consolidation agent. This is expected to stabilize the wood and prevent future degradation of the wood. A modest pH environment will also contribute to keep TBDMS chitosan stable over time.

Besides acidity, the presence of metal ions—iron in particular—is a well-known and widespread threat for archaeological wood. The chitosan molecule’s ability to chelate metal ions makes it to a promising study object in research on new, bio-inspired, and multifunctional conservation materials. Further research is required to investigate if the modified chitosan retains the ability of untreated chitosan to trap metal ions and to bind to protons. The solubility of TBDMS chitosan in toluene and ethyl acetate shows promise to use the material as an alternative consolidant for archaeological wood that does not tolerate treatments using aqueous solvents.
